# Macrophage inflammatory factors promote epithelial-mesenchymal transition in breast cancer

**DOI:** 10.18632/oncotarget.24917

**Published:** 2018-03-23

**Authors:** Robert B. Bednarczyk, Neha Y. Tuli, Elyse K. Hanly, Ghada Ben Rahoma, Rachana Maniyar, Abraham Mittelman, Jan Geliebter, Raj K. Tiwari

**Affiliations:** ^1^ Department of Microbiology and Immunology, New York Medical College, Valhalla, NY, USA

**Keywords:** epithelial-mesenchymal transition, inflammation, M1 macrophages, metastasis, secretory factors

## Abstract

The majority of breast cancers (90–95%) arise due to mediators distinct from inherited genetic mutations. One major mediator of breast cancer involves chronic inflammation. M1 macrophages are an integral component of chronic inflammation and the breast cancer tumor microenvironment (TME). Previous studies have demonstrated that up to 50% of the breast tumor comprise of tumor-associated macrophages (TAMs) and increased TAM infiltration has been associated with poor patient prognosis. Furthermore, breast cancer associated deaths are predominantly attributed to invasive cancers and metastasis with epithelial-mesenchymal transition (EMT) being implicated. In this study, we investigated the effects of cellular crosstalk between TAMs and breast cancer using an *in vitro* model system. M1 polarized THP-1 macrophage conditioned media (CM) was generated and used to evaluate cellular and functional changes of breast cancer lines T47D and MCF-7. We observed that T47D and MCF-7 exhibited a partial EMT phenotype in the presence of activated THP-1 CM. Additionally, MCF-7 displayed a significant increase in migratory and invasive properties. We conclude that M1 secretory factors can promote a partial EMT of epithelial-like breast cancer cells. The targeting of M1 macrophages or their secretory components may inhibit EMT and limit the invasive potential of breast cancer.

## INTRODUCTION

Breast cancer is the most common malignancy in women within the United States affecting around 1 in 8 women [1–2] with an estimated number of breast cancer cases in 2018 to be 268,670 [1]. Breast cancer is also the second leading cause of cancer-related deaths in females with approximately 40,450 attributed deaths and is second only to lung cancer [1]. These high death rates can be primarily attributed to invasive breast cancers that metastasize and form secondary tumors [3]. Increased breast screenings, newer aggressive therapeutics, and a reduction in hormone replacement therapy has resulted in a decline in breast cancer deaths since early 2000; however, metastatic breast cancer is still a major challenge [4, 5].

Around 5–10% of total breast cancer occurrences are a result of inherited genetic mutations (e.g., BRCA1/2), which leaves an astounding 90–95% of breast cancers that develop due to the influence of outside mediators. One major mediator of breast cancer development and progression is inflammation. Inflammation is a natural biological process that occurs during times of infection or wound healing and is self-limiting which is hence called acute inflammation. During chronic inflammation this self-limiting process is deregulated and can contribute to a number of diseases including: Alzheimer's [6], cardiomyopathy [7], osteoarthritis [8], inflammatory bowel disease (IBD) [9], and numerous types of cancers including breast [10–12]. Specifically, chronic inflammation has been associated with cancer initiation and progression [13–15].

One significant constituent of chronic inflammation is macrophages which are also a main resident of the breast tumor microenvironment (TME). Macrophages that reside within the TME are termed tumor-associated macrophages (TAMs) and have been shown to comprise up to 50% of the total tumor mass in certain breast cancer clinical cases with poor prognosis associated with increased TAM tumor infiltration [16–18].

Macrophages are subdivided into two distinct phenotypes, M1 or M2, depending on specific stimuli including cytokines and bacterial moieties. M1 macrophages are classically activated and pro-inflammatory and have traditionally been deemed as anti-tumor due to their ability to generate reactive nitrogen and oxygen intermediates, and pro-inflammatory cytokines [19, 20]. On the contrary, M2 macrophages are alternatively activated and anti-inflammatory, promoting tumor progression by downregulating the immune response and stimulating angiogenesis [21, 22]. Although M1 macrophages are known to have anti-tumor properties, these immune cells enhance tumor development and progression through frequent tissue damage and disruption in addition to pro-inflammatory cytokines secreted.

Epithelial-mesenchymal transition (EMT) is a cellular process where epithelial cells lose adhesive properties and cell polarity to become motile and mesenchymal-like. Although EMT is known to be a natural occurrence serving important roles in development [23] and wound healing [24], cancer cells can harness this process in ultimately enhancing metastasis. Currently, the roles of M1 macrophages influencing breast cancer cells to undergo EMT is not widely known or studied.

Our laboratory has previously demonstrated that estrogen treated murine mammary breast cancer, and human thyroid cancer cells, secrete factors that promote human umbilical vein endothelial cell (HUVEC) migration and tube formation. These factors are contained within the conditioned media generated from these cancer cells. [25, 26]. In this study, we used a similar methodology of generating conditioned media and investigated the role of M1 macrophage secretory factors and their potential in inducing EMT in epithelial-like breast cancer cell lines, T47D and MCF-7. We determined that a human monocytic cell line, THP-1, when polarized towards an M1 phenotype can induce breast cancer cells to undergo a partial EMT; thereby, having the potential to stimulate breast cancer metastasis.

## RESULTS

### Activation of THP-1 with TPA induces an M1 macrophage phenotype

**Figure 1:**
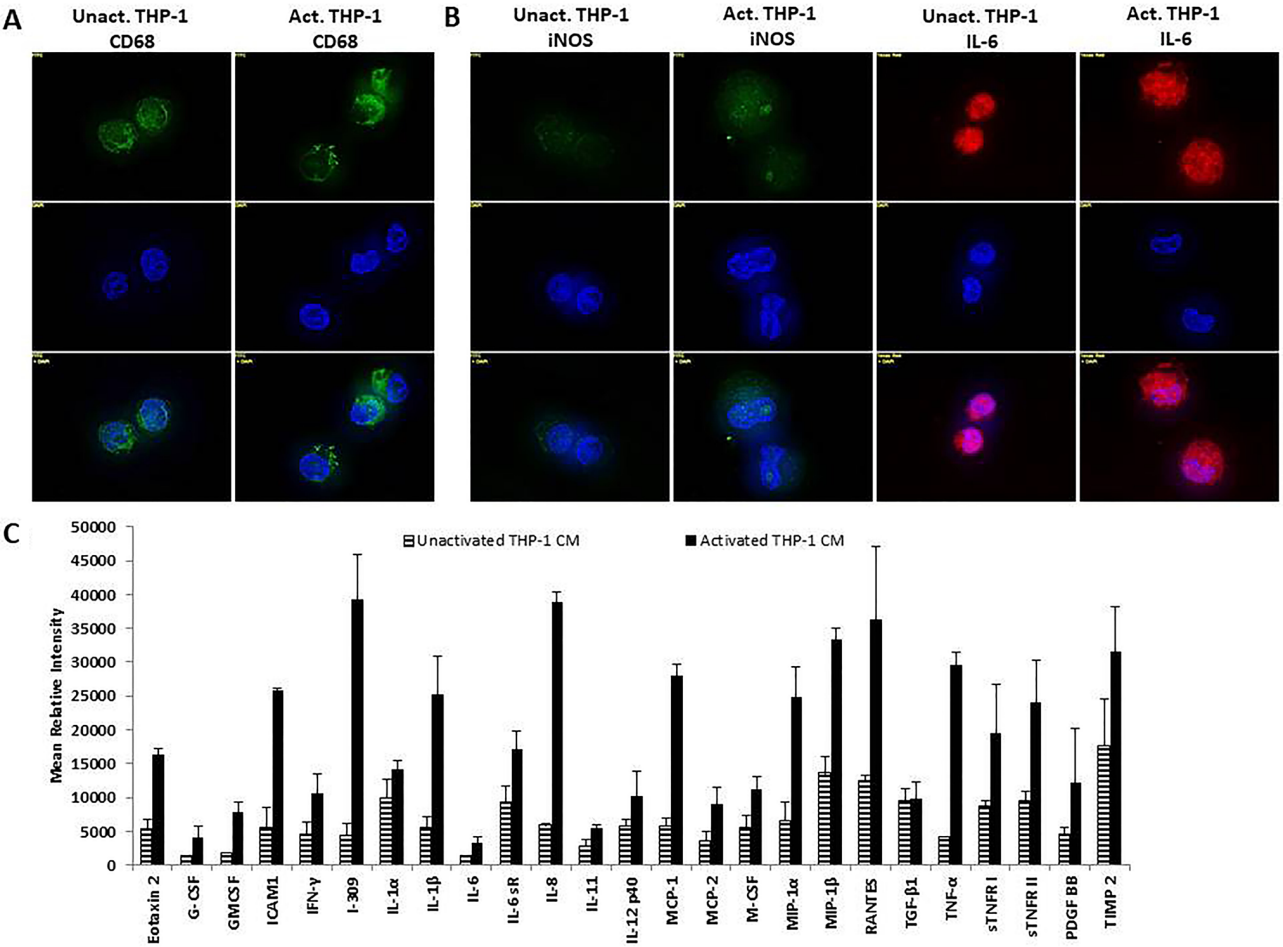
THP-1 cells exhibit an M1 macrophage phenotype following activation with TPA (**A**) THP-1 cells express monocyte/macrophage lineage marker CD68. (**B**) Upon activation with TPA, THP-1 cells express M1 macrophage markers iNOS and IL-6. Unactivated and activated THP-1 cells were seeded overnight and probed with CD68, iNOS, or IL-6 primary antibodies. Fluorescent secondary antibodies were used for immunofluorescence. Pictures were taken using Axiovision Rel 4.8 at 100x magnification. (**C**) Activated THP-1 cells express pro-inflammatory cytokines including: TNF-α, IL-1β, and IL-8. A human inflammation array was performed on unactivated or activated THP-1 CM (48 h). Cytokine/chemokine blots were analyzed using ImageJ and optical density determined and normalized ± SEM of two independent experiments.

In order to determine the effects of cellular crosstalk between TAMs and breast cancer mediated by macrophage secretory factors, the macrophage phenotype of THP-1 monocytes activated with 12-O-Tetradecanoylphorbel-13-acetate (TPA) was first identified. Unactivated and activated THP-1 were probed for CD68, a transmembrane protein which is a common monocyte/macrophage lineage marker. Both, unactivated and activated, THP-1 cells expressed CD68 validating this cell type being of monocyte/macrophage lineage (Figure 1A). M1 macrophage markers iNOS and IL-6 were also evaluated using immunofluorescence analysis. Activated THP-1 cells expressed increased iNOS and IL-6 compared to unactivated THP-1 cells (Figure 1B). Additionally, an analysis of the cytokine profile of activated THP-1 cells showed a pro-inflammatory cytokine expression of TNF-α, IL-1β, and IL-8 (Figure 1C). This indicates that THP-1 cells, upon activation with TPA, promotes an M1 macrophage phenotype which is a major contributor to chronic inflammation.

### Activated THP-1 secretory factors alter breast cancer cell morphology and reduces viability

**Figure 2:**
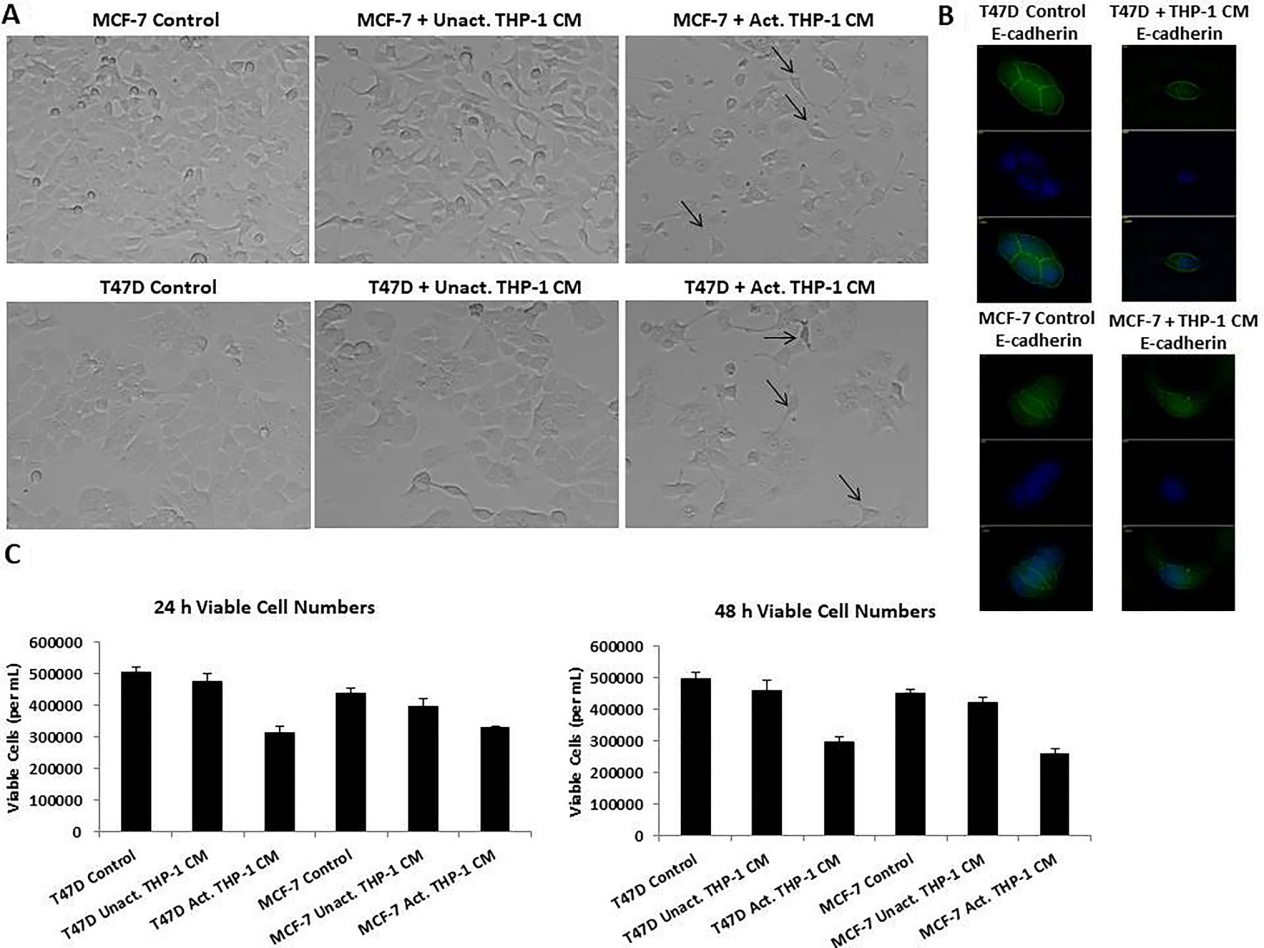
T47D and MCF-7 cells exhibited spindle-like morphology, decreased E-cadherin, and decreased viability viable cells following treatment with activated THP-1 CM (**A**) Treatment of T47D and MCF-7 with activated THP-1 CM promotes mesenchymal-like morphological changes. Breast cancer cells were treated with unactivated or activated THP-1 CM for 24 h and cell morphology was observed by phase contrast microscopy. Pictures were taken at 20x magnification using phase contrast microscopy. (**B**) T47D and MCF-7 were treated for 24 h with control (5% CS-RPMI) or activated THP-1 CM and probed with E-cadherin primary antibodies. Fluorescent secondary antibodies in addition to DAPI (nuclear stain) were used for immunofluorescence. Pictures were taken using Axiovision Rel 4.8 at 100x magnification. (**C**) Activated THP-1 CM leads to decreased number of viable T47D and MCF-7 cells at 24 and 48 h. A trypan blue exclusion assay was performed to evaluate the number of viable cells. Data represents the number of viable cells in duplicates ± SEM of two independent experiments.

In order to evaluate the effects of activated THP-1 secretory factors on breast cancer phenotype, we used an *in vitro* model system; T47D and MCF-7 human breast cancer cells treated with unactivated or activated THP-1 conditioned media (CM). T47D and MCF-7 human breast cancer cells are of the luminal subtype being estrogen receptor positive and Her2 negative and are presumably amenable to epithelial-mesenchymal transition. These cell lines in their native state in cell culture are epithelial-like. Activated THP-1 CM induced spindle-like morphology of breast cancer cells compared to unactivated THP-1 CM or control treatments (Figure 2A). T47D and MCF-7 are epithelial-like cells and a transition to spindle-like morphology, indicates a change in cellular phenotype in breast cancer cells, resembling mesenchymal cells. There was decreased E-cadherin expression in T47D and MCF-7 following activated THP-1 CM treatment (Figure 2B). A trypan blue exclusion assay was used to determine the number of viable breast cancer cells following THP-1 CM treatments. We observed a significant decrease in the number of viable T47D and MCF-7 following 24 h treatment with activated THP-1 CM (Figure 2C).

**Figure 3:**
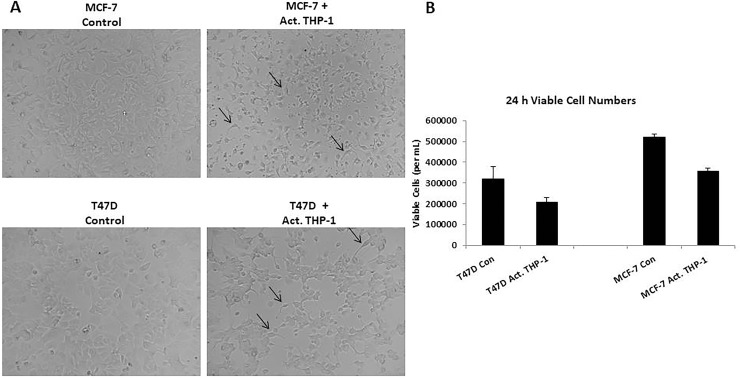
Spindle-like morphology and reduced viability is observed following co-culture with activated THP-1 cells (**A**) Co-culture of activated THP-1 cells with either T47D or MCF-7 promotes mesenchymal-like morphology. Breast cancer cells were co-cultured for 24 h and cell morphology was observed. Pictures were taken at 10x magnification using phase contrast microscopy. (**B**) A decreased number of viable breast cancer cells was observed following co-culture with activated THP-1 cells for 24 h. A trypan blue exclusion assay was performed to evaluate viability. Data represents the number of viable cells in duplicates ± SEM of two independent experiments in duplicates.

In order to evaluate cellular interactions between M1 macrophages and breast cancer cells, we performed co-culture experiments with activated THP-1 and breast cancer cell lines using 0.4-μM transwell chambers. We evaluated cell morphology and the viability. As observed with activated THP-1 CM treatments, T47D and MCF-7 exhibited spindle-like morphology (Figure 3A) and a significant decrease in the number of viable cells (Figure 3B) in the presence of activated THP-1 cells. Thus, activated THP-1 secretory factors are promoting a mesenchymal-like phenotype in addition to decreased cellular growth of breast cancer cells.

### M1 macrophage inflammatory factors upregulate epithelial-mesenchymal transition related transcription factors

**Figure 4:**
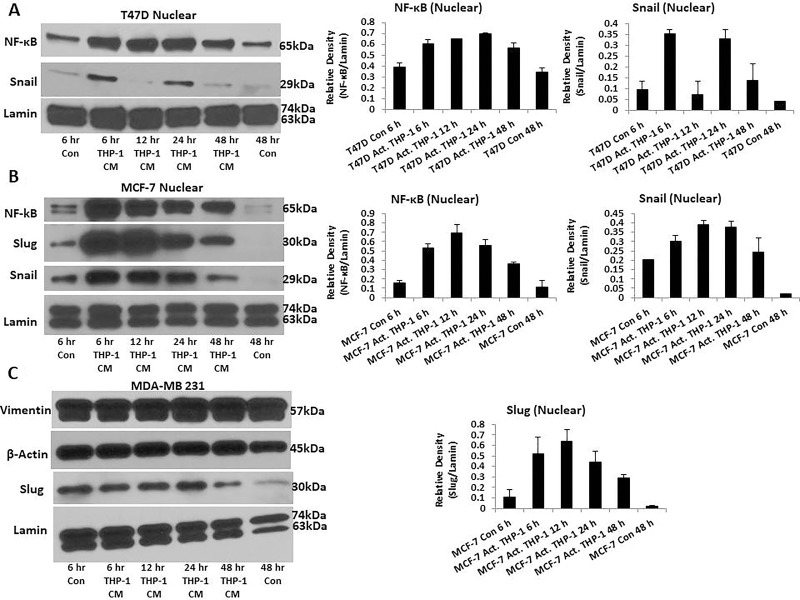
T47D and MCF-7 cells exhibit increased nuclear localization of EMT transcription factors (**A**) Increased nuclear localization of NF-κB and Snail is observed in T47D. (**B**) MCF-7 displays increased nuclear localization of NF-κB, Snail, and Slug. (**C**) MDA-MB 231 robustly expresses mesenchymal markers Vimentin and Slug. T47D, MCF-7, and MDA-MB 231 were treated with activated THP-1 CM for 6 h, 12 h, 24 h, and 48 h. Nuclear extractions were prepared and Western blots performed probing for: NF-κB (65 kDa), Slug (30 kDa), Snail (29 kDa), Vimentin (57 kDa), β-Actin (45 kDa), and Lamin (63, 74 kDa). Relative density of NF-κB, Snail, and Slug is shown normalized to Lamin loading controls ± SEM of two independent experiments.

Spindle-like morphology and reduced viability are prominent characteristics of cells undergoing EMT. Therefore, we evaluated whether activated THP-1 secretory factors are promoting the EMT process. We assessed EMT transcription factors and markers in T47D and MCF-7 treated with activated THP-1 CM using Western blot analysis and immunofluorescence. We observed increased nuclear localization of EMT transcription factors NF-κB and Snail in T47D (Figure 4A), while MCF-7 displayed increased nuclear localization of NF-κB, Snail, and Slug (Figure 4B). MDA-MB 231 that is considered post-EMT was used for comparison and displays robust mesenchymal Vimentin and Slug expression throughout (Figure 4C). Therefore, M1 macrophage secretory factors induce EMT transcription factor and marker changes.

### M1 macrophage inflammatory factors promote MCF-7 migration and invasion in addition to increased MMP-9 expression

**Figure 5:**
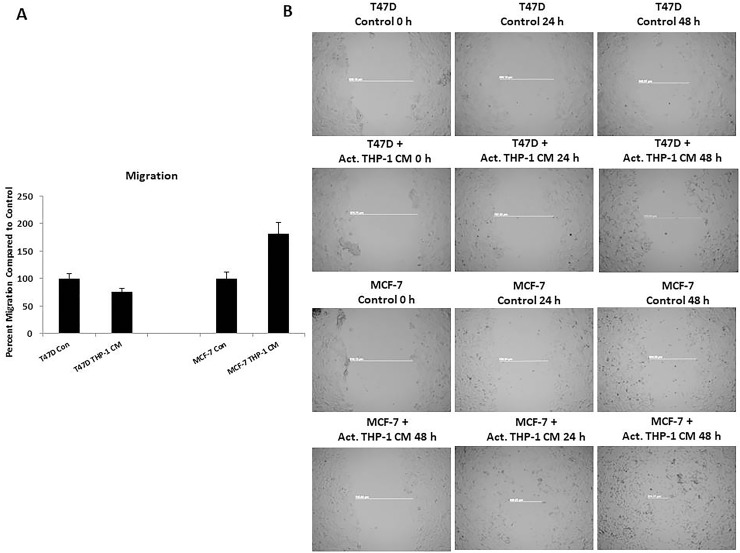
THP-1 CM promotes MCF-7 migration (**A**) MCF-7 exhibits increased migration at 18 h in the presence of activated THP-1 CM. Breast cancer migration was evaluated using Boyden chamber assays. Pictures were taken using brightfield microscopy at 5x magnification counting four fields per insert. Data is represented as percent migration compared to control in duplicates ± SEM of two independent experiments. (**B**) Scratch wound assay confirms increased MCF-7 migration. Scratch-wound assay was performed on T47D and MCF-7 treated with control (5% CS-RPMI) or activated THP-1 CM with mitomycin C (2 μg/mL) for 24 h. Pictures were taken at 0 h, 24 h, and 48 h following control or CM treatments using phase contrast microscopy and Axiovision Rel 4.8 at 5x magnification.

**Figure 6:**
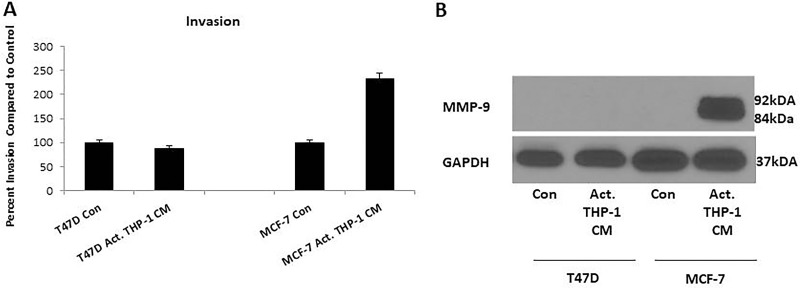
Activated THP-1 CM treatment enhances MCF-7 invasion and MMP-9 expression (**A**) MCF-7 exhibits increased invasion at 18 h in the presence of activated THP-1 CM. Breast cancer invasion was evaluated using Boyden chamber assays. Pictures were taken using brightfield microscopy at 5x magnification counting four fields per insert. Data is represented as percent invasion compared to control in duplicates ± SEM of two independent experiments. (**B**) There is increased expression of MMP-9 by MCF-7 with 24 h activated THP-1 CM treatment. MMP-9 (84, 92 kDa) and GAPDH (37 kDa) expression was evaluated using Western blots.

Since there was increased nuclear localization of EMT transcription factors and decreased E-cadherin expression on breast cancer cells, induction of EMT was indicative. We evaluated migration and invasion of T47D and MCF-7 following activated THP-1 CM treatments since these processes are known to be upregulated by cells undergoing EMT. Breast cancer cell migration and invasion was evaluated using a Boyden chamber assay. Following an 18 h treatment with activated THP-1 CM, MCF-7 exhibited a significant increase in migration and invasion compared to untreated controls (Figure 5A, Figure 6A). Additionally, a scratch wound assay confirmed an increase in MCF-7 migratory capability in the presence of activated THP-1 CM (Figure 5B). T47D did not express significant changes in either migration or invasion following similar treatments (Figure 5A, Figure 6A).

Cells that have increased migratory and invasive capabilities are known to secrete matrix metalloproteinases (MMPs) such as MMP-2 and MMP-9 which breakdown the extracellular matrix allowing cells to migrate and invade tissues [27, 28]. We thus evaluated MMP-9 expression in breast cancer cells following activated THP-1 CM treatments via Western blot analysis. We observed increased MMP-9 expression in MCF-7 that correlated with the increased migration and invasion as previously shown (Figure 6B). Additionally, since there were no observed changes in migration or invasion for T47D with M1 secretory factors, we did not detect changes in MMP-9 (Figure 6B). Therefore, increased MMP-9 expression may contribute to the migratory and invasive potential of MCF-7.

## DISCUSSION

Chronic inflammation is known to contribute to several types of diseases including cancer. One integral component of chronic inflammation involves immune cells, specifically macrophages. Macrophages are divided into M1 or M2 phenotypes and both types have been implicated in cancer development or progression. Anti-inflammatory cytokines such as IL-4, IL-13, and TGF-β may polarize macrophages into M2 phenotypes which are considered immunosuppressive and can contribute to breast cancer progression by promoting growth, metastasis, and angiogenesis. On the contrary, IFN-γ, GM-CSF, and LPS can polarize macrophages into M1 phenotype. Since the predominant role of M1 macrophages is related to inflammation and that chronic inflammation has a detrimental role in cancer progression, this study was focused on M1 macrophage based cellular transition. M1 macrophages are pro-inflammatory and have been traditionally deemed as having anti-tumor activity. However, M1 macrophages through chronic inflammation have been associated with cancer initiation due to introducing tissue damage [19, 20, 29] and cytokine secretion [19, 30, 31]. In addition, the TME of numerous types of cancers including breast contain TAMs. Therefore, in this study we investigated the influence of M1 macrophages on breast cancer cells through cellular crosstalk mediated by macrophage secretory factors. We determined that cellular crosstalk mediated by M1 secretory factors promoted EMT of luminal subtypes of breast cancer.

We used an *in vitro* model system to evaluate the interactions between infiltrating macrophages and breast cancer as would resemble within the TME. Previous studies have demonstrated that human monocytic cell lines such as THP-1 when activated with TPA exhibit an M1 phenotype [32–34]. We generated CM and observed that following the activation of THP-1 with TPA, there was enhanced expression of M1 marker iNOS in addition to pro-inflammatory cytokines IL-6, TNF-α, IL-1β, and IL-8 when compared to unactivated THP-1.

To evaluate cellular crosstalk mediated by pro-inflammatory macrophage secretory factors on breast cancer cells, we examined the cellular and functional changes caused by activated THP-1 CM on luminal/epithelial-like breast cancer cell lines T47D and MCF-7. We observed that activated THP-1 secretory factors induced EMT-like features in breast cancer cells. T47D and MCF-7 exhibited mesenchymal-like morphology and decreased viability following activated THP-1 CM and co-culture experiments. In addition, there was increased nuclear localization of EMT-related transcription factors for T47D and MCF-7. EMT-related transcription factors have functional roles in downregulating epithelial markers such as E-cadherin which is a component of adherens junctions and disruption of this protein promotes increased migratory capability of cells [35, 36]. We evaluated E-cadherin expression in breast cancer cells following activated THP-1 CM treatment. There was a loss of E-cadherin in spindle-like T47D and MCF-7 cells following treatments. Decreased E-cadherin expression would subsequently influence breast cancer cell motility warranting an inquiry into cell migration and invasion.

Cell migration and invasion are processes that are identified as being upregulated in cells undergoing EMT. We observed that activated THP-1 CM promoted MCF-7 to demonstrate significantly increased migration as observed through Boyden chamber and scratch wound assays. Additionally, there was increased invasion and MMP-9 expression by MCF-7. T47D did not display changes in migration or invasion in the presence of activated THP-1 CM compared to control. As was previously shown, T47D displayed an increase in Snail nuclear localization which was observed to be oscillatory through 48 h. The irregular nuclear localization of Snail and the absence of Slug may have negatively affected T47D migratory and invasive properties as opposed to MCF-7 in which Snail and Slug expression was dynamic. Therefore, MCF-7 displayed a more progressed EMT compared to T47D.

Several studies have formerly established a link between inflammatory factors and cancer EMT [37–40]. Pro-inflammatory cytokines such as TNF-α have been previously demonstrated to induce EMT in a number of cancers including breast cancer [41, 42]. TNF-α has been shown to stimulate the induction of EMT transcription factors including Snail, Slug, Twist, and ZEB1/2 through NF-κB signaling [41, 43]. It is possible that TNF-α which is secreted by activated THP-1 promotes the induction of EMT as observed in T47D and MCF-7. Further studies examining the neutralization of TNF-α in activated THP-1 CM and the effects on EMT induction should be carried out.

Other cytokines that have been implicated in EMT and were present in activated THP-1 CM include: IL-1β [44], IL-6 [45], IL-8 [46] and TGF-β [47]. Additionally, the influence of macrophage secreted exosomes containing miRNA cargo was not evaluated. Exosomes are small (30-120 nm) membraneous nanovesicles that are secreted from cells and have been implicated in cell-cell communication [48]. Depending on the cell type, exosomes can contain various types of cargo including miRNA. Several studies have reported that miRNA can contribute to EMT [49, 50]. Therefore, it is possible that macrophage secreted exosomes containing particular miRNA may promote EMT as observed with T47D and MCF-7.

We demonstrate that pro-inflammatory secretory factors can induce a partial EMT in breast cancer cells, displaying a combination of epithelial and mesenchymal characteristics. Additionally, certain breast cancer cell lines may be more prone to undergoing EMT compared to others. In our study, we observed that MCF-7 exhibited increased EMT properties compared to T47D following similar treatments. Breast cancer cells that undergo EMT have a greater likelihood of contributing to metastasis, decreasing survival outcomes for patients. Additionally, breast cancer cells that have been shown to undergo EMT may not only contribute to increased metastasis, but may become resistant to cytotoxic T lymphocytes [51]. Therefore, the degree of tumor cell EMT can potentially affect how a patient may or may not respond to immunotherapy which has an important implication in patient treatment. Further it should be noted that epithelial-mesenchymal transition has inherent plasticity which is well integrated with stem cell population generation, [52] and has considerable redundancies as all factors, mediators and signal transducers are not induced concurrently.

We propose that by directly targeting M1 macrophages or neutralizing pro-inflammatory secretory factors, the chronic inflammatory response may be limited and potentially reduced ultimately hindering breast cancer progression. By reducing breast cancer invasive potential through the inhibition of EMT processes, the risk of metastasis decreases and the prospect of patient survival increases. A better understanding of the cells found within the TME and how they impact breast cancer progression will help in terms of breast cancer therapy.

## MATERIALS AND METHODS

### Cells and cell culture

The breast cancer cell lines used include: T47D and MCF-7 which were obtained from American Type Culture Collection (ATCC). T47D and MCF-7 cells were grown in RPMI-1640 (Mediatech, Herndon, VA) and DMEM-1640, respectively, supplemented with 10% fetal bovine serum (FBS) (Atlanta Biologicals, Atlanta, GA), penicillin 10,000 IU/mL, streptomycin 10,000 μg/mL (Mediatech) and 2 mM L-glutamine (Mediatech). Human monocytic cell line, THP-1, was grown in RPMI-1640 (Mediatech, Herndon, VA), supplemented with 10% FBS (Atlanta Biologicals, Atlanta, GA), penicillin 10,000 IU/mL, streptomycin 10,000 ug/mL (Mediatech), 2 mM L-glutamine (Mediatech), and 0.05 mM 2-mercaptoethanol. T47D, MCF-7, and THP-1 cell lines were purchased between 2000 to 2013 and were authenticated by the Genomics Core at Albert Einstein School of Medicine using SoftGenetics GeneMarkerHID.

### Differentiation of THP-1 and generation of conditioned media

THP-1 monocytes were seeded at a density of 5×10^6^ cells and treated with 200 nM 12-O-Tetradecanoylphorbel-13-acetate (TPA) in RPMI-1640 supplemented media for THP-1 as specified above. The media was discarded and rinsed with phenol red free RPMI-1640 following incubation for 48 h. THP-1 cells were further incubated for 48 h in 5% charcoal-stripped RPMI to obtain THP-1 macrophage conditioned media (CM). The conditioned media was then collected and centrifuged for 5 min at 1500 rpm to remove cells and cell debris. Conditioned media was stored in -80°C until future use.

### Morphology analysis

T47D and MCF-7 were seeded in 6 well plates at 3×10^5^ per well in duplicates and allowed to adhere and grow overnight in RPMI or DMEM media as previously described, respectively. Seeding media was removed from the cell monolayer and rinsed with phenol-red free RPMI. Breast cancer cells were treated with either 5% charcoal-stripped phenol-red free RPMI as control, unactivated THP-1 CM, or activated THP-1 CM for CM evaluation for 24 h. Pictures were taken under phase contrast microscopy at 20× magnification.

### Trypan blue exclusion assay

T47D and MCF-7 were seeded in 6 well plates at 3 × 10^5^ per well in duplicates and allowed to adhere and grow overnight in RPMI or DMEM culture media as previously described, respectively. Seeding media was removed from the cell monolayer and rinsed with phenol-red free RPMI. Breast cancer cells were treated with either 5% charcoal-stripped phenol-red free RPMI, unactivated THP-1 CM, or activated THP-1 CM for 24 or 48 h. Following respective time points, cells were harvested and stained using 0.4% trypan blue (Sigma Chemical). Viable (unstained) cells were counted using a hemocytometer.

### Co-culture experiments

THP-1 were activated using TPA for 48 h and seeded in 0.4-μm transwell 6-well plates (Corning^TM^ Costar^TM^) at 1 × 10^6^ cells per well in duplicates. T47D and MCF-7 were seeded in 6 well plates at 3 × 10^5^ cells per well in duplicates and allowed to adhere and grow overnight in RPMI or DMEM culture media as previously described. Seeding media was removed from the cell monolayer and rinsed with phenol-red free RPMI. Activated THP-1 were rinsed with phenol-red free RPMI. Breast cancer cells were incubated with 5% charcoal-stripped phenol-red free RPMI. 0.4-μm transwells for controls and activated THP-1 contained 5% charcoal-stripped phenol-red free RPMI and respective co-cultures transpired for 24 h. Morphology pictures were taken using phase contrast microscopy at 10× magnification. Viable cells were determined using trypan blue exclusion assay as mentioned above.

### Western blot analysis of total cell or subcellular fractionated lysates

The generation of total cell lysates or subcellular fractionated lysates was prepared by two different methods.

### Total cell lysates

Following activated THP-1 CM treatments, breast cancer cells were harvested and lysates generated using RIPA Lysis Buffer (50 mM Tris-HCL pH 7.5, 150 mM NaCl, 1% NP-40, 0.5% Sodium deoxycholate, 0.1% SBS) with Halt^TM^ Protease and Phosphatase Inhibitor Cocktail (1X Concentration) (Life Technologies). Membranes were blocked with 5% milk in TBST (10 mM Tris-HCL, pH 7.5, 200 mM NaCl, 0.05% Tween-20) for 2 h at room temperature and then incubated overnight in primary antibodies: MMP-9 (Cell Signaling) and GAPDH (Cell Signaling). Membranes were washed 3 times for 10 min per wash and incubated with secondary antibodies of goat anti-rabbit IgG (H+L) Horseradish Peroxidase (Thermo Scientific) for 2 h at room temperature.

### Subcellular fractionated lysates

Following activated THP-1 CM treatments, breast cancer cells were harvested and cytoplasmic and nuclear protein extracts generated using NE-PER Nuclear and Cytoplasmic Extraction Reagents (Thermo Scientific) as per manufacturer's instruction with Halt^TM^ Protease and Phosphatase Inhibitor Cocktail (1X Concentration) (Life Technologies). Membranes were blocked with 5% milk in TBST (10 mM Tris-HCL, pH 7.5, 200 mM NaCl, 0.05% Tween-20) for 2 h at room temperature and then incubated overnight in primary antibodies of either: NF-κB (Cell Signaling), Snail (Cell Signaling), Slug (Cell Signaling), and Lamin A/C (Cell Signaling) at 4°C. Membranes were washed 3 times for 10 min per wash and incubated with secondary antibodies of either goat anti-rabbit IgG (H+L) Horseradish Peroxidase (Thermo Scientific) or rabbit anti-mouse IgG (H+L) Horseradish Peroxidase (Thermo Scientific) for 2 h at room temperature.

### Migration and invasion assays

BD Biocoat Control Inserts (BD Biosciences) and BD Biocoat Matrigel Invasion Chambers (BD Biosciences) with 8-μm pore membrane filters were used for the migration and invasion assays, respectively. Matrigel inserts were rehydrated for 2 h, 37°C with phenol-red free RPMI. Inserts were then incubated with 2.5 × 10^4^ cells/0.5 mL of T47D or MCF-7 in the presence of 5% charcoal-stripped phenol-red free RPMI (control) or 48 h activated THP-1 CM with 10% charcoal-stripped RPMI in the chamber as the chemoattractant. Following 18 h treatments at 37°C, 5% CO_2_ atmosphere, the removal of non-migrating/invading cells on the upper surface was performed through scraping with a cotton swab. Cells were fixed with 100% methanol for 2 min and stained with 1% Toluidine Blue in 1% Borex for 2 min. Excess stain was removed through rinsing the inserts in distilled water and allowed to dry overnight. Pictures were taken using PixeLINK Megapixel FlatWire Camera Release 3.2 on the Axiovert 200 M microscope (Carl Zeiss MicroImaging, Inc., Thornwood, NY) at 5x magnification counting four fields per insert.

### Scratch-wound assay

Migration was assessed for T47D and MCF-7 using a scratch-wound assay. Cells were seeded in 6 well plates at 5 × 10^5^ cells per well in duplicates in RPMI or DMEM media as previously described. Cells were allowed to adhere and grow to 60–75% confluent cell monolayers. Two vertical wounds were created using a 10 μL sterile pipette tip followed by removal of detached cells and cell debris by rinsing with phenol-red free RPMI. The wounded cell monolayer was treated with either 5% charcoal-stripped phenol-red free RPMI or activated THP-1 CM with mitomycin C (2 μg/mL) for 24 h. Phase contrast microscopy captured pictures at 0 h and 24 h following treatments using Axiovision Rel 4.8 on the Axiovert 200 M microscope (Carl Zeiss MicroImaging, Inc., Thornwood, NY) at 5× magnification.

## Abbreviations

CM: conditioned media
EMT: epithelial-mesenchymal transition
MMPs: matrix metalloproteinases
TPA: 12-O-Tetradecanoylphorbel-13-acetate
TAMs: tumor-associated macrophages
TME: tumor microenvironment
